# Patients’ perspectives and the perceptions of healthcare providers in the treatment of early rectal cancer; a qualitative study

**DOI:** 10.1186/s12885-023-11734-0

**Published:** 2023-12-21

**Authors:** Lisanne J. H. Smits, Annabel S. van Lieshout, Saskia Debets, Sacha Spoor, Leon M. G. Moons, Koen C. M. J. Peeters, Stefan E. van Oostendorp, Olga C. Damman, Rien J. P. A. Janssens, Wytze Lameris, Nicole C. T. van Grieken, Jurriaan B. Tuynman

**Affiliations:** 1 Department of Surgery, Amsterdam UMC, Vrije Universiteit Amsterdam, Cancer Centre Amsterdam, De Boelelaan 1117, Amsterdam, 1081HV the Netherlands; 2 Department of Gastroenterology, University Medical Center Utrecht, Utrecht, the Netherlands; 3 Department of Surgery, Leiden University Medical Center, Leiden, the Netherlands; 4 Department of Surgery, Rode Kruis Ziekenhuis, Beverwijk, the Netherlands; 5 Department of Public and Occupational Health, Vrije Universiteit Amsterdam, Public Health Research Institute, Amsterdam, the Netherlands; 6 Medical Research Ethics Committee Brabant, Tilburg, the Netherlands; 7 Department of Pathology, Amsterdam UMC, Vrije Universiteit Amsterdam, Cancer Centre Amsterdam, Amsterdam, the Netherlands

**Keywords:** Early rectal cancer, Patients’ perspectives, Organ preservation

## Abstract

**Background:**

Shared decision-making has become of increased importance in choosing the most suitable treatment strategy for early rectal cancer, however, clinical decision-making is still primarily based on physicians’ perspectives. Balancing quality of life and oncological outcomes is difficult, and guidance on patients’ involvement in this subject in early rectal cancer is limited. Therefore, this study aimed to explore preferences and priorities of patients as well as physicians’ perspectives in treatment for early rectal cancer.

**Methods:**

In this qualitative study, semi-structured interviews were performed with early rectal cancer patients (*n* = 10) and healthcare providers (*n* = 10). Participants were asked which factors influenced their preferences and how important these factors were. Thematic analyses were performed. In addition, participants were asked to rank the discussed factors according to importance to gain additional insights.

**Results:**

Patients addressed the following relevant factors: the risk of an ostomy, risk of poor bowel function and treatment related complications. Healthcare providers emphasized oncological outcomes as tumour recurrence, risk of an ostomy and poor bowel function. Patients perceived absolute risks of adverse outcome to be lower than healthcare providers and were quite willing undergo organ preservation to achieve a better prospect of quality of life.

**Conclusion:**

Patients’ preferences in treatment of early rectal cancer vary between patients and frequently differ from assumptions of preferences by healthcare providers. To optimize future shared decision-making, healthcare providers should be aware of these differences and should invite patients to explore and address their priorities more explicitly during consultation. Factors deemed important by both physicians and patients should be expressed during consultation to decide on a tailored treatment strategy.

**Supplementary Information:**

The online version contains supplementary material available at 10.1186/s12885-023-11734-0.

## Background

Over the past years, patient engagement in clinical decision-making has gained importance [1–3]. Several steps can be identified in shared decision-making. First, the patient needs to be informed on the available options and their respective benefits and harms [3–7]. Next, the patient, in dialogue with the physician, ideally expresses individual values and preferences. In the final step both patient and physician participate in clinical decision-making [3–7]. Despite the broad variety of literature and great number of guidelines available on shared decision-making, there often is a lack of insight in patients’ perspectives in specific diseases or disease stages, such as early rectal cancer [8, 9]. Perhaps as a result, clinical decision-making is still frequently based on physicians’ perspectives [10, 11].

For patients with rectal cancer, several treatment options are available. Standard treatment for rectal cancer is total mesorectal excision (TME) surgery combined with (neo)adjuvant (chemo)radiotherapy according to the stage of the disease [12]. These treatment modalities aim to decrease the risk of local tumour recurrence, and implementation of these techniques has led to an improvement in oncological outcomes [13]. However, radical surgery is associated with complications, ostomy rates, functional bowel complaints, sexual- and micturition problems, which contribute to a deterioration in quality of life [14–16]. Consequently, there is a growing interest in organ preservation to omit TME and the risk of an ostomy [17]. In rectal cancer, local excision of the tumour combined with (neo)adjuvant (chemo)radiotherapy has the potential to save the rectum. In this strategy, patients do not require an ostomy and have a lower risk of functional bowel complaints, sexual- and micturition problems. Unfortunately, organ preservation presumably leads to a higher risk of local tumour recurrence, which may cause insecurity or anxiety in patients. A meta-analysis showed a local recurrence rate of 4% after completion surgery compared to 14.7% after adjuvant chemoradiotherapy in locally excised pT2N0 tumours [18]. Consequently, organ preservation requires frequent follow-up examinations to timely diagnose potential recurrences. In clinical decision-making, deliberating between these two treatment options results in a challenging balance in which oncological outcomes need to be weighed against morbidity and prospects of quality of life.

There is little insight into patients’ perspectives regarding priorities and treatment preferences in early rectal cancer  [19]. Therefore, this qualitative study aimed to identify factors that early rectal cancer patients deem important in their treatment and compare patients’ perspectives to healthcare providers’ perceptions.

## Methods

### Design & procedure

**Fig. 1:**
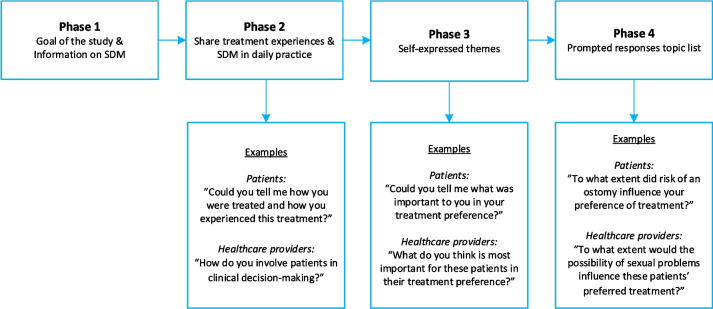
Flow of the interview

This qualitative study involved a single semi-structured interview with early rectal cancer patients and healthcare providers. Based on the limited available literature and experience from healthcare providers in the research team, a topic list for the interviews was designed and pilot tested [20]. The final version of the topic list is available in Supplementary Material 1. Through an iterative process the topics physical condition and work- and leisure activities were added to the list during the study. The interviews were conducted by two female researchers (LS and AvL), both medical doctors currently working as PhD-students in the field of early rectal cancer. The interviewers were supervised by senior researchers in the field of early rectal cancer as well as by senior researchers in qualitative methods. The interviewers had no pre-existing relationships with participants. More detailed information regarding the interviewers and reflexivity is provided in Supplementary Material 2. The interview was divided into several parts (Fig. 1). Patients were initially informed on the goal of the study and the essence of shared decision-making. In the first part of the interview, patients were asked to explain their involvement in the decision-making process and to share how they experienced their treatment. Next they were asked to elaborate on the factors that had influenced their priorities and preferences by open-ended questions. For example: “Could you tell me what was important to you in your treatment preference?”. The final part of the interviews consisted of specific questions to obtain prompted responses regarding a list of selected factors (i.e. tumour recurrence, survival, risk of an ostomy, risk of treatment related complications, bowel function, sexual problems, micturition problems, pain, frequency of follow-up and uncertainty associated with treatment). For instance:”To what extent did the risk of an ostomy influence your preference of treatment?” In interviews with healthcare professionals, these questions were converted to the perspective of healthcare providers. First, healthcare providers were asked about how they inform patients and how they apply shared decision-making in daily practice. Further questions aimed to obtain insight in what healthcare providers perceived that patients would deem important in their treatment. For example: “What do you think is most important for these patients in their preference of treatment?” or “To what extent would the possibility of sexual problems influence these patients’ preferred treatment?”. To gain further insight and to investigate the interrelationship of the discussed topics, patients and healthcare providers were asked to rank the items included in the topic list, listing these items from number 1, being the most important, to number 10, being the least important. Participants were asked to perform the ranking after completion of the interview, to prevent the interview from being directed towards the ranked items.

### Setting & participants

Patients from one tertiary colorectal care centre (Amsterdam University Medical Centre, location VU medical centre) who were previously treated for early rectal cancer and were informed of or participated in the TESAR trial (NCT02371304) were recruited. The TESAR trial is an ongoing randomised controlled trial that investigates the long-term oncological outcomes of adjuvant chemoradiotherapy and completion TME after local excision of high-risk pT1 and low-risk pT2 cancers [21]. Thereby, all participants received additional study consultation and explanation of the potential benefits and risks of both rectum preservation and TME surgery. Since local excision followed by chemoradiotherapy is still experimental, only patients who participate in clinical trials can undergo this type of treatment in the Netherlands. For that reason, inclusion of patients who deliberately chose this type of treatment was not possible and patients who participated in a randomised trial had to be selected. Exclusion criteria included language barriers and legal incapacity. Patients were invited if their next follow-up appointment was within three months from the study invitation letter and if their traveling distance was less then forty kilometres to the Amsterdam University Medical Centre, location VU medical centre. Patients were informed of the study via a letter and telephone call. A convenient sample size of ten patients were invited to participate in the study. This sample size was based on an estimation of the number of patients needed to reach data saturation. The invited patients consisted of equal numbers of sex and cancer treatment. No patients with end ostomies met the inclusion criteria. Only, patients with temporary ostomies could be invited. A similar sample size of ten healthcare providers was invited as well. Healthcare providers were invited if they either were involved in the multidisciplinary team meetings of colorectal cancer care in the Amsterdam University Medical Centre, location VU medical centre or if they have been actively participating in the TESAR trial. Interviews with patients were conducted and analysed prior to the interviews with healthcare providers. The study was approved by the investigational research board of the Amsterdam University Medical Centre, location VUmc (2021.0147). Written informed consent was obtained from all participants.

Patient interviews were performed at the outpatient clinic or at the patient’s home according to their preference, allowing a comfortable environment and optimizing in-depth conversations. In three patients a spouse or first degree family member was present during the interview. Healthcare providers were interviewed at the hospital, by video conference or over the phone. Inclusion of participants continued until no new topics, preferences or influencing factors were identified and data saturation was achieved. Interviews were performed between June 2021 and February 2022. During the interviews imposed precautions and measures of the Dutch government against COVID-19 were followed. Nonetheless, all patients interviews could have been held in person, whereas some healthcare providers had to be interviewed over the phone or by video conference. All interviews were recorded by an audio recorder and converted to transcripts (LS and AvL). Transcripts were not returned to participants for comments.

### Analyses

The transcribed interviews were independently analysed and coded by two researchers (LS and AvL) using ATLAS.ti9 (Version 9.0.22.0). Thematic analysis approach was used to analyse the data [22]. First, the reviewers familiarised themselves with the data by reading the transcriptions thoroughly. Second, the open coding methodology was performed to break down and analyse the data [22, 23]. Next, comparison of initial coding progressed by linking the data using axial coding and after consensus was reached final relationships and themes were developed. Patient data were analysed prior to interviews and analyses of data of healthcare providers. In this iterative process, the factors physical condition and work and leisure activities were added to the topic list after analyses of the first patients’ data. Analyses were separated between patients and healthcare providers and at first separate codebooks were developed. During the axial coding phase, roughly the same themes could be developed for both patients and healthcare providers. The ranked items were sorted by the lowest median scores and interquartile ranges for both patients and healthcare providers. Data were reported according to the consolidated criteria for reporting qualitative research (COREQ) (Supplementary material 3) [24]. Strategies to enhance rigour were recorded in Supplementary material 4 [25].

## Results

### Participants’ characteristics

In total, ten patients and ten healthcare providers were interviewed. Baseline characteristics of these groups are depicted in Tables 1 and 2. Of the patients, four (40%) were females. Median age was 68.5 (range 59 – 86) years old. The duration of the interviews varied from 43 to 163 min. Four patients underwent local excision followed by completion surgery, four received local excision followed by adjuvant chemoradiotherapy and two underwent local excision only. Data saturation was reached after interviewing nine patients. Of the invited sample size, patient 5, a 86 year old male with comorbidities was excluded, because he felt that local excision as a sole treatment was the only treatment option that was discussed. Therefore, he found it hard to debate other treatment options and the respective consequences. The interviewed healthcare providers consisted of three colorectal surgeons, two gastroenterologists, two radiotherapists, one oncologist, one surgical physician assistant and one surgical resident. Five (50%) healthcare providers were females. Since only a small number per subspecialty of healthcare providers was invited, all interviews were performed and analysed, even though data saturation was reached after eight interviews. The duration of the interviews varied from 22 to 38 min.

**Table 1:** Characteristics of patients

**Participant**	**Sex**	**Treatment**	**Ostomy**	**Follow-up**^**a**^**(months)**	**Education**^**b**^
1	Female	CRT	No	46	Bachelor or equivalent
2	Male	TME	Temporary	34	Bachelor or equivalent
3	Female	CRT	No	29	Short-cycle tertiary education
4	Male	TME	No	29	Bachelor or equivalent
5	Male	WW	No	24	Master or equivalent
6	Female	TME	Temporary	21	Bachelor or equivalent
7	Male	CRT	No	12	Bachelor or equivalent
8	Female	TME	Temporary	7	Bachelor’s degree or equivalent
9	Male	CRT	No	5	Post-secondary non-tertiary education
10	Male	WW	No	5	Upper secondary

*CRT* chemoradiotherapy, *TME* total mesorectal excision, *WW* watchful waiting local excision only

^a^Time between treatment and interview

^b^Education level according to the international standard classification of education 2011

**Table 2:** Characteristics of healthcare providers

**Participant**	**Sex**	**Position**	**Experience (years)**
1	Male	Gastroenterologist	14
2	Male	Gastroenterologist	8
3	Female	Radiotherapist	7
4	Male	Radiotherapist	17
5	Female	Oncologist	4
6	Male	Colorectal surgeon	15
7	Female	Colorectal surgeon	1
8	Male	Colorectal surgeon	10
9	Female	Surgical resident	5^a^
10	Female	Physician assistant	4^b^

^a^Years since start of residency

^b^Years working as a physician assistant

### Self-reported factors

**Fig. 2:**
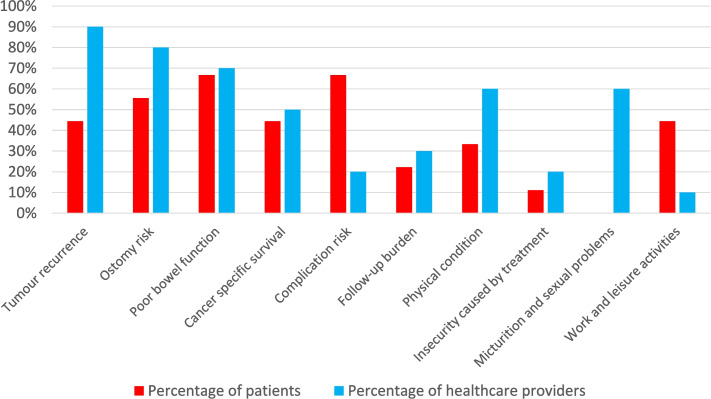
Self-reported themes expressed spontaneously during the first part of the interview

During the first part of the interview, patients were asked about the factors that they deemed important in their treatment. Healthcare providers were asked about the factors they thought patients would find the most important. Figure 2 demonstrates the self-reported themes that were expressed during the first part of the interviews. The frequency in which these topics are expressed may be indicative for importance. Patients frequently mentioned the risk of complications, preservation of bowel function and the risk of an ostomy, whereas the factors tumour recurrence, risk of an ostomy and preservation of bowel function were often expressed by healthcare providers. Perspectives of both patients and healthcare providers on major themes are illustrated in Table 3.

**Table 3:** Major themes and perspectives

**Theme**	**Representative patients’ quotes**	**Representative healthcare providers’ quotes**
Bowel function	“I thought maybe I will be held back by having to go to the toilet ten to twelve times a day. So I would not be able walk the dog for an hour or so. Or I would not be able to go out of the house. I would be crazy to want that.” [patient 9]	“I do not think they immediately dwell on the thought now I go regularly and later on four to five times a day. I do not think that is on their mind.” [healthcare provider 4]
	“I could not really assess it beforehand. I could not imagine what it would be like. […] I thought it would just be some stomach cramps, or going to the toilet a bit more frequently. I could not have imagined that it would be present throughout the day.” [patient 6]	“Patients often wonder what it will be like after surgery. How often do I need to go to the toilet? Will I be incontinent? If I’m in the car will I be able to stop in time? That is really on their mind, bowel function.” [healthcare provider 8]
Ostomy	“What does it mean when you have an ostomy, it must have a big impact on your life. Look, eventually you will learn to live with it, but the initial phase will still be a lot of hard work.” [patient 1]	“Of course, patients are reluctant to have an ostomy, are afraid so to speak. But that is mostly the association they have with it, a bag of poop.” [healthcare provider 1]
Tumour recurrence	“In the end it is all about probabilities, debating these probabilities is something you have to do yourself. What are my chances of recurrence, what if it goes wrong and can I live with that outcome.” [patient 4]	“What strikes me is that when patients actually hear the numbers on the risk of recurrence and lymph node metastases, that they often think well that is not too bad. While we (healthcare providers) are making a big fuss about a few percent more or a few percent less.” [healthcare provider 1]
		“I think that most patients think, one percent of additional risk is already a lot when we are discussing cancer.” [healthcare provider 2]
		“Oncological outcomes are the most important” [healthcare provider 10]
Survival	“No, life is too beautiful to take risks.” [patient 4]	“I think they just generally care about surviving the cancer.” [healthcare provider2]
	“I never thought of that (the risk of dying), that did not influence my decision.” [patient 10]	
Treatment related complications	“I didn't ask about the complications of the operation in detail because the doctor told me that they had gained experience with this operation. The doctor told me: we can do it well, you shouldn't underestimate it, but we can do it well. So it didn't sound so threatening. And for the radiation, I actually hadn't heard that much about complications. Those were not stressed and therefore I assumed it wasn't that bad either.” [patient 8]	“It depends on the information that is given. But if you tell them what can happen during an operation, they always say: oh dear that is a very big operation. So I think they debate the risk of complications.” [healthcare provider 6]
		“I find that difficult, to physicians it is extremely important. But patients often tell me: of course I understand that surgery is not without risks. I don’t know if it influences their decision-making.” [healthcare provider 1]
Work and leisure activities	“And on top of that I have a pottery next door, I taught. I lived alone, a lot of practical problems would come my way when I would have an operation.” [patient 3]	
	“I took complete care of my husband […] and because of that I actually didn’t have a lot of time to recover and I thought with surgery I am in the hospital for a while.” [patient 1]	

### Impact of treatment on daily life & treatment burden

Most patients found it very important that treatment and its consequences would fit in with their daily life. If the type of treatment was not compliant with factors as work or leisure activities, many patients felt that such an option would cause burden and would negatively impact their quality of life. For these reasons some patients expressed that they preferred organ preservation. For example:

*“My wife and I are full of life and I still want to do a lot of stuff. I thought if after surgery I would need to go to the bathroom 10–12 times a day, I would feel held back. To not be able walk the dog for an hour or so, I would be crazy to want that.” [patient 9].*

In addition, few patients said to have based their preference on contextual factors such as being a primary caregiver or taking care of pets. In line with these findings, some healthcare providers expressed that social factors, such as sports, shopping and social gatherings, could influence treatment preference. Moreover, healthcare providers linked this theme to patients’ physical condition. After the first interviews questions regarding physical condition and work and leisure activities were included in the topic list. The majority of healthcare providers thought that a reduction in physical condition could influence treatment preferences in patients. More specifically, in elderly patients loss of physical condition caused by radical completion surgery could lead to treatment burden and a loss of quality of life. Accordingly, some of the patients declared that they weighed the impact of treatment on their physical condition in their preference.

Another potential burden expressed by healthcare providers were the frequently required follow-up examinations and appointments associated with organ preservation. Healthcare providers thought that this may be a reason for patients to prefer completion surgery. In contrast, several patients reported appreciation for frequent follow-up and all patients expressed that the reassurance of good results and additional tests for them outweighed the stress and time investment in these examinations.

In addition, most healthcare providers shared that the increased risk of cancer recurrence in organ preservation potentially causes burden in patients through experienced anxiety or insecurity. However, patients’ opinions concerning this subject differed. Some patients interpreted the risk of recurrence of organ preservation to be low, whereas one patient spontaneously expressed that this risk and subsequent insecurity led him to prefer completion surgery. Healthcare providers felt that the influence of insecurity depends on the patient’s personality and explained that during consultations, they as professionals try to estimate the impact of this factor on the well-being of individual patients.

### The importance of preserving bowel function

The majority of patients expressed that preservation of bowel function and the chances of low anterior resection syndrome (e.g. symptoms as incontinence, soiling, urgency, clustering, increased frequency of stool and emptying difficulties) played a key role in their preference (Table 3). Patients reasoned that poor bowel function would impact their quality of life. In the second part of the interview all but one patient mentioned that bowel function was important to them. For instance, one patient described the following:

*“Upon further questioning, it turned out that surgery might cause a decline in bowel function. Yes, and that frightened me to such an extent that I thought is there no other way.” [patient 9].*

Some patients added that they themselves appreciated a holistic view and the integrity of the body and the rectum. The continuity of the rectum, its functions and a do no harm principle were important to these patients. Therefore, they did not prefer completion surgery. Furthermore, two patients remembered that it was hard to assess the implications of bowel problems during counselling. These statements were in accordance with reports of healthcare providers. For example one radiotherapist stated:

“*Also, beforehand people cannot estimate what kind of role that (bowel dysfunction) plays. How much discomfort and burden it causes. […] The whole social burden, I think that is not properly assessed.” [healthcare provider 3].*

In general, both patients and healthcare providers agreed that bowel function is one of the most important factors that should guide decision-making, because of the impact of poor bowel function on daily life.

### Ostomy risks are of great importance to patients due to a bad reputation

Another aspect prioritized by both patients and physicians was the risk of an ostomy. Only one patient, without an ostomy, mentioned an ostomy in a neutral context. She stated:

*“If you do need an ostomy, then you are going to have to live with that. It is a matter of guidance, accepting and learning how to handle it. You can still move on with your life.”*

Other patients typically placed ostomies in a negative context (Table 3). For example:

*“Getting an ostomy was my biggest concern. Fortunately, that was not the case, which was quite a relief. […] An ostomy would turn my life upside down.” [patient 4]*

The comments of healthcare providers were in line with the reports of patients (Table 3). One surgeon stated:

*“Some patients may be quite willing to take some oncological risks (tumour recurrence) to avoid an ostomy.” [healthcare provider 6]*

Furthermore, one surgeon added:

*“I think that they do not know exactly what it (an ostomy) is, they think that you cannot do anything with it, that you always have leakage. Yes those are just horror stories that are still told.” [healthcare provider 7].*

Those “horror stories” referred to by this surgeon indeed emerged from the patient interviews, since patients often illustrated distinctly negative experiences that they had heard from family, friends or acquaintances. Due to these stories, they thought an ostomy would impact their daily activities and quality of life. In contrast to the perspectives of the interviewed patients, some healthcare providers expressed that some patients can be relieved to get an ostomy after a long period of bowel function problems, however, these examples generally referred to cases of inflammatory bowel disease.

### The risk of tumor recurrence appraised differently by patients and healthcare providers

Patients appeared to be aware of the trade-off to be made between the risk of local recurrence and quality of life when discussing potential organ preservation treatment strategies (Table 3). Some patients thought of surgery as a “back-up treatment”, meaning that if the tumour would reoccur the next step would be to undergo radical surgery. Since they felt a back-up was available they preferred to try organ preservation first. Nevertheless, patients interpreted the risks of local recurrence to be relatively low. For example, one patient stated:

*“It is still taking a risk, but for me the risk was just very small actually.” [patient 7]*

In addition, patients expressed to have been reassured by the fact that the tumour had been completely removed during local excision:

*“It (the tumour) was small and it was already completely removed during the first operation. […] It (completion surgery) was more precautionary, a larger piece had to be removed to prevent it (the tumour) from metastasizing.” [patient 8].*

Healthcare providers seemed to perceive the relative risk of tumour recurrence to be higher than patients and believed that the risk of local recurrence would be the most important factor to patients (Table 3). For example:

*“I think the most important factor ultimately is oncological outcome (tumour recurrence and cancer related survival).” [healthcare provider 9]*

These assumptions are also reflected by the large majority of healthcare providers that identified tumour recurrence as a factor that would significantly influence patients during clinical decision-making.

### Cancer related survival seems less important to patients in early rectal cancer

Even though several patients identified survival as a factor that should be incorporated in decision-making, only one patient explicitly stated in the interview that the risk of dying ultimately played a role in his preference (Table 3). All other patients, explained that the risk of dying because of cancer did not play a role in their own preferences or priorities, mostly because they thought that the absolute risk of dying from early rectal cancer was very low. (Table 3). One patient recalled:

*“For example pancreatic cancer, that is different, only ten per cent or less survives that. For bowel and rectal cancer we looked up the numbers on the internet. I do not even remember the exact percentages. Anyway, the survival rates are so high, I never thought this was life-threatening.” [patient 9].*

Opposed to the reports of patients, the majority of healthcare providers thought that cancer specific survival was an influencing factor (Table 3). An illustration:

*“The recurrence rate is of course very important to patients, and cancer progression. I think that is important during counselling. Also survival. Just the hard outcome measures.” [healthcare provider 4].*

### Perspectives on treatment related complications differ

The possibility of complications was often reported by patients during the first part of the interview, yet the influence of complications on their preference varied among them. A few patients were concerned about complications of chemoradiotherapy and some patients described that postoperative complications specifically played a role in their treatment preference. One patient stated:

*“That does play into your head. Will I be okay? Will I get a complication after surgery? And those are all things that make it difficult to make a decision.” [patient 6]*

Others took complications for granted and merely thought of treatment related complications as a part of the preferred treatment. For instance:

*“Why worry about that beforehand? If something happens you have to cope with it. You simply have to live with it.” [patient 3]*

For complications related to chemoradiotherapy particularly, some patients felt that those complications depended on how their body would react to treatment. A similar variation in importance and perceived preferences of patients could be observed among healthcare providers (Table 3). Moreover, a few patients noted that the way they thought about complications was mostly based on the manner physicians presented and discussed treatment related complications. An example is provided in Table 3.

### Sexual and micturition problems are important to healthcare providers

Sexual or micturition problems were self-reported factors identified by most healthcare providers. Patients on the other hand did not report that these factors contributed to their preference. If asked specifically about these problems, patients explained that they were informed on these topics, yet they either thought risks were low or felt that it was not applicable to their situations due to other circumstances (e.g. for sexual function: no partner or pre-existing problems in sexual function).

### Ranking of items

To gain more insight in the different perspectives and the interrelationship of the previously described themes, items on the topic list were ranked by patients and healthcare providers based upon importance in potential decision-making. Even though participant numbers were small and heterogeneity may be present, some differences in ranking between patients and healthcare providers may be noted. Individual ranking outcomes and median ranking scores of both patients and healthcare providers are depicted in Tables 4 and 5. Patients found the risk of an ostomy, bowel function and treatment related complications to be the most important (Table 4). Healthcare providers ranked the risk of an ostomy highest, followed by tumour recurrence and bowel function (Table 5). Healthcare providers seemed to rank oncological outcome measures higher than patients. For example, in contrast to healthcare providers ranking tumour recurrence second tumour recurrence was ranked fourth by patients. Moreover, treatment related complications were ranked sixth by healthcare providers compared to third by patients.

**Table 4:** Ranking of themes by patients

	**#1**	**#2**	**#3**	**#4**	**#6**	**#7**	**#8**	**#9**	**#10**	**Median score (IQR)**
Ostomy risk	8	2	1	3	8	1	1	2	1	2 (1 – 3)
Preserving bowel function	7	1	2	6	1	2	2	3	2	2 (2 – 3)
Treatment related complications	1	3	3	2	2	3	3	1	4	3 (2 – 3)
Tumour recurrence	3	4	5	1	3	5	5	7	3	4 (3 – 5)
Insecurity burden	4	5	6	5	4	4	6	5	7	5 (4 – 6)
Micturition problems	5	7	4	7	6	10	4	4	5	5 (4 – 7)
Frequency of follow-up	9	8	10	8	7	9	10	6	6	8 (7 – 9)
Cancer related survival	2	10	7	4	9	7	8	8	9	8 (7 – 9)
Pain	6	6	8	9	10	6	7	10	10	8 (6 – 10)
Sexual problems	10	9	9	10	5	8	9	9	8	9 (8 – 9)

**Table 5:** Ranking of themes by healthcare providers

	**#1**	**#2**	**#3**	**#4**	**#5**	**#6**	**#7**	**#8**	**#9**	**#10**	**Median score (IQR)**
Ostomy risk	5	4	2	2	2	1	2	2	1	2	2 (2 – 2)
Tumour recurrence	2	2	6	3	4	2	1	4	5	1	2.5 (2 – 4)
Preserving bowel function	3	7	3	1	3	4	3	1	8	4	3 (3 – 4)
Cancer related survival	1	1	1	6	1	3	8	8	10	6	4.5 (1 – 7.5)
Insecurity burden	4	5	5	4	5	5	5	5	2	5	5 (4.25 – 5)
Treatment related complications	6	3	10	8	7	10	6	3	3	3	6 (3 – 7.75)
Micturition problems	7	9	8	5	6	8	4	10	7	9	7.5 (6.25 – 8.75)
Sexual problems	8	8	4	10	8	9	7	6	6	8	8 (6.25 – 8)
Frequency of follow-up	10	10	9	7	10	6	9	7	4	7	8 (7 – 9.75)
Pain	9	6	7	9	9	7	10	9	9	10	9 (7.5 – 9)

## Discussion

This qualitative study of nineteen semi-structured interviews with patients and healthcare givers provides an overview of factors that were deemed important in treatment of early rectal cancer. The study identified that the risk of an ostomy, poor bowel function, tumour recurrence, and treatment related complications were important themes to both patients and healthcare providers. Patients seemed to consider themes that would impact their daily activities and quality of life as most important, while healthcare providers seemed to focus on oncological outcomes, such as tumour recurrence and cancer specific survival. This may be valuable knowledge for healthcare providers when applying shared decision-making in daily practice.

In general, healthcare providers seemed to be aware that the impact of surgery on quality of life causes patients to often prefer organ preservation. Still our results showed that, compared to healthcare providers, patients were more willing to trade oncological safety to avoid an ostomy, functional problems or surgery-related complications. The observed difference between healthcare providers and patients may potentially be even more substantial in non-academic hospitals, in which less experience may be present in organ preservation for rectal cancer. Only limited data is available regarding the differences in perspectives between patients and healthcare providers in colorectal cancer care [26–28]. Nonetheless, these studies also suggested that rectal cancer patients, compared to physicians, are more willing to sacrifice oncological outcomes to avoid a deterioration in quality of life [26–28]. These described differences in perspectives of patients and healthcare providers seem to be underestimated by clinicians and could influence clinical- and shared decision-making in daily practice.

In spite of the complex treatment options in rectal cancer and the observed differences, there is little guidance for clinicians on how to inform rectal cancer patients appropriately and how to help patients to form a preference based on their own values [8, 9, 29, 30]. To support patients in forming treatment preferences several decision aids have been developed in the field of colorectal cancer [29, 31, 32]. These decision aids, however, do not focus on early stages of the disease, but discuss surgery type or chemotherapy in more advanced disease stages. A systematic review by Hommes et al*.* on decision aids in stage I-III of colorectal cancer only included one decision aid that aimed at organ preservation in patients with a clinical complete response after radiotherapy [31]. In this decision aid the provided information seems to align more with the observed perspectives of healthcare providers in the current study than the perspectives of patients [33]. Moreover, Hommes et al*.* reported that available decision aids lack possibilities of including personalised information and that the overall communicative quality of decision aids in colorectal cancer is low, which reduces the applicability of these aids in daily practice [31]. Previous studies in other fields did show that the use of decision aids by patients before and during consultation leads to improved knowledge, more accurate risk perceptions, greater clarity of values and a more active role in decision-making [34–36]. Similar results were found in a study that evaluated a decision aid for surgery type in rectal cancer patients, which observed improved knowledge and a reduction of decisional conflict in patients [32]. Besides, in the current study some healthcare providers did suggest that in early rectal cancer treatment information provided to patients varies among physicians and expressed that synchronisation of information may be beneficial to support patients in forming their preferences. Consensus on the information provided to patients during consultation should be established, for example by a Delphi study including both patients and healthcare providers. A potential solution to support both patients and clinicians may lie in a well-developed education tool and decision aid that includes personalised comprehensive treatment information, as well as questions about patient’s values and daily activities to help patients gain insight into their personal treatment preferences and may ultimately help patients to engage in clinical decision-making [8]. Such tools could also substantially synchronize and improve communication by healthcare providers and could provide clinicians guidance in shared decision-making.

This qualitative study has limitations. First, a sample size of twenty participants may not reflect the perspectives of all rectal cancer patients and healthcare providers. Also, themes that emerged from interviews with healthcare providers were roughly similar to topics included in the topic list, whereas in patient interviews themes as physical condition, work, leisure activities and impact on daily life were identified. Similarities between the topic list and themes identified in interviews with healthcare providers may have been caused by the fact that the topic list was partially developed through discussion between healthcare providers in the field of early rectal cancer. Nevertheless, major factors emerged consistently throughout the interviews and data saturation was reached in both groups. Second, since interviews were held after treatment, decision regret or post-hoc justification may have influenced outcomes. Moreover, since participating patients were frequently referred from local hospitals to be informed on organ preservation or had asked for organ preservation themselves, it may be presumed that health literacy in participants was high and perspectives of underserved groups were represented less [37]. In addition, participants that are currently facing early rectal cancer may have been able to express their preferences in greater detail compared to participants that need to remember how they felt during this period. Another limitation is the potential bias that was introduced by not being able to include patients with permanent ostomies. None of the patients that met the inclusion criteria had to receive a permanent ostomy, therefore only patients with temporary ostomies could be included. A study population that included patients with end ostomies would have been preferable and the lack of these patients may impact the external validity of this study. Moreover, the study only included patients with early rectal cancer which could explain why patients not deemed cancer specific survival as important as expected, given the lower risk of cancer progression in the early disease stage. Last, all interviewed patients were informed on or participated in the TESAR trial [21], a randomized clinical trial that compares total mesorectal excision with adjuvant chemoradiotherapy after local excision for early stage rectal cancer. Therefore, the included patients could not make a true treatment decision. Local excision followed by chemoradiotherapy is an experimental type of treatment and can only be provided in randomised trials in the Netherlands. Also the selected study population implies that included patients were at least interested in organ preservation, which might introduce bias. Prior to treatment patients were informed about organ preservation by their treating physician. Consequently, the provided information may have differed among patients and the time between treatment and the interview varied which may have influenced outcomes. Since rectal preservation strategies are still in development and robust evidence of oncological safety is lacking. It is yet unknown if rectum preservation strategies such as local excision combined with (neo)adjuvant (chemo)radiotherapy are oncologically safe. Long-term results of large randomized clinical trials such as the TESAR trial [21] and STAR-TREC trial [38] are awaited and will support clinical decision-making. This knowledge gap concerning oncological safety may contribute to the challenges in communication with patients.

## Conclusions

In conclusion, this study explored patients’ and healthcare providers’ perspectives on treatment of early rectal cancer. Patients’ preferences in treatment of rectal cancer vary and frequently differ from the assumptions and beliefs of healthcare providers. Overall, patients seemed to be quite willing to sacrifice oncological outcomes to maintain their daily activities and to gain a better prospect of quality of life. Clinicians should be aware of their assumptions of patients’ preferences and should assure themselves that they foster dialogue on patients’ preferences, values and daily activities. The development of training in shared decision-making, an educational tool and decision-aid could improve shared decision-making, by providing guidance to clinicians and supporting patients to express priorities and preferences.

### Supplementary Information

**Additional file 1: Supplementary material 1.** Topic list patients.

**Additional file 2: Supplementary material 2.** Characteristics of researchers that performed interviews and data analyses.

**Additional file 3: Supplementary material 3.** COREQ (COnsolidated criteria for REporting Qualitative research) Checklist.

**Additional file 4: Supplementary material 4.** Strategies to achieve rigour^25^.

## Data Availability

The datasets used and/or analysed during the current study are available from the corresponding author on reasonable request.

## Abbreviation

TME: Total mesorectal excision
