# Supplementary material for: Patients’ perspectives and the perceptions of healthcare providers in the treatment of early rectal cancer; a qualitative study
Source: BMC Cancer. 2023 Dec 21;23:1266. doi: 10.1186/s12885-023-11734-0 (PMC10740344; doi:10.1186/s12885-023-11734-0)
Supplement: Supplementary file 1 — Additional file 1: Supplementary material 1. Topic list patients. [file 12885_2023_11734_MOESM1_ESM.docx]

**Supplementary material 1. Topic list patients.**

The topic list is based on patients. Healthcare providers will be asked about what they perceive that patients would deem important during clinical decision-making. If topics are discussed during the first part of the interview, these topics will be explored at that time.

**General introduction**

*When were you treated for early rectal cancer?*

*Can you elaborate on how that treatment went?*

In this interview, we want to focus on factors you deemed important when debating treatment options. It does not matter which option ultimately had your preference, but we would like to know more about how you came to your preference.

*How did you make the choice to participate in the trial at the time?*

*Can you tell us more about that?*

*What was important in your choice to participate at the time?*

*Which factors played a role in your treatment preference?*

*Did you look up certain information or asked questions to your health care providers?*

*Why were you interested in this information?*

*Why did you want to know more about it?*

We would also like to know more about your experiences with the decision-making process and the role of information and caregivers.

*Did you feel involved in the decision-making process?*

*Why yes / no, can you explain?*

*On a scale of 1-10, can you indicate the extent to which you felt involved?*

*Why do you choose this rating?*

*Did you feel satisfied about the extent of involvement?*

During the second part of the interview patients were questioned about specific topics. In addition, they were be asked which topics were not important to them and why these topics were not important.

**Local tumour recurrence**

*Did local tumor recurrence play a role for you?*

*To what extent?*

*Did you feel that this risk was low or high?*

**Cancer specific survival**

*Did the possibility of dying from the disease play a role for you?*

*To what extent?*

*Does it matter to your consideration whether this is a low probability or a high probability?*

**Ostomy**

*Did the possibility of a stoma play a role for you?*

*To what extent?*

*If an ostomy was required, would it have been a temporary or an end ostomy?*

*Do you have a temporary or end ostomy?*

**Likelihood of complications from additional treatment**

*Did the risk of complications play a role for you?*

*To what extent?*

*Did you experience any complications during your treatment?*

*Which ones?*

*What was necessary to resolve them?*

**Pain**

*Did the possibility of pain play a role for you?*

*To what extent?*

*Did you experience pain during your treatment?*

*Did you receive pain medication?*

**Bowel function**

*Did future problems with defecation play a role for you?*

*To what extent?*

*Do you currently have more frequent bowel movements, thinner stools, more difficulty delaying defecation, more frequent involuntary loss of stool than before your treatment?*

*What does this mean to you?*

*How are you dealing with these problems?*

**Micturition problems**

*Did potential future problems with urination play a role for you?*

*To what extent?*

*Are you currently having micturition problems?*

**Sexual function**

*Did potential sexual problems play a role for you?*

*To what extent?*

*Are you currently having sexual problems?*

**Frequency of follow-up**

*Did the frequency of follow-up examinations and appointments play a role for you?*

*To what extent?*

*Would you find it inconvenient to undergo frequent follow-up examinations and appointment or not?*

*Why or why not?*

**Uncertainty**

*Did uncertainty about tumour recurrence play a role for you?*

*To what extent?*

*Does it matter to your consideration whether this is a low probability or a high probability?*

*How are you currently dealing with this?*

**The following topics were added to the topic list during the iterative process.**

**Physical condition**

*Did your physical play a role for you?*

*To what extent?*

*Did a potential decline in you physical condition play a role for you?*

*To what extent?*

**Work and leisure activities**

*Did your work play a role for you?*

*To what extent?*

*Did any social or leisure activities play a role for you?*

*To what extent?*

**Closure**

*Are there any factors that we have not yet discussed that that you would like to discuss?*

*Which factors were most important for you in your treatment?*

*Can you indicate an order the factors that we have discussed?*
