# Supplementary material for: Patients’ perspectives and the perceptions of healthcare providers in the treatment of early rectal cancer; a qualitative study
Source: BMC Cancer. 2023 Dec 21;23:1266. doi: 10.1186/s12885-023-11734-0 (PMC10740344; doi:10.1186/s12885-023-11734-0)
Supplement: Supplementary file 2 — Additional file 2: Supplementary material 2. Characteristics of researchers that performed interviews and data analyses. [file 12885_2023_11734_MOESM2_ESM.docx]

| **Characteristics at time of interviews** | **Researcher 1 (LS)** | **Researcher 2 (AvL)** |
| --- | --- | --- |
| Sex | Female | Female |
| Age | 29 | 26 |
| Background | Medical doctor, PhD-student | Medical doctor, PhD-student |
| Clinical experience | Two years of clinical experience in surgery | One year of clinical experience in surgery |
| Research experience | Two years of experience in clinical research particularly in early rectal cancer | One years of experience in clinical research in both early rectal cancer and transanal total mesorectal excision |
| Relationship with participants prior to study commencement | None. Researcher was not part of the treatment team | None. Researcher was not part of the treatment team |
| Participants knowledge of the interviewer | Basic. The only information provided was a short introduction of the researcher and that she performed research in the field of early rectal cancer | Basic. The only information provided was a short introduction of the researcher and that she performed research in the field of early rectal cancer |
| Expectations prior to interviews | Patients would probably prefer organ preservation, mainly due to the idea of an ostomy. Nevertheless, they would feel a lot of insecurity prior to follow-up and might even experience fear | Patients would probably prefer organ preservation, since the chance of cancer recurrence sounds low to them, and they do not wish for an ostomy. In addition, they probably are not able to properly assess the consequences of recurrence |
| Personal preference if faced with early rectal cancer | Surgery, because of age and risk averse personality traits | Chemoradiotherapy, because (the chance of) low anterior resection syndrome and an ostomy would decrease my quality of life |

**Supplementary material 2. Characteristics of researchers that performed interviews and data analyses.**
