# Supplementary material for: Patients’ perspectives and the perceptions of healthcare providers in the treatment of early rectal cancer; a qualitative study
Source: BMC Cancer. 2023 Dec 21;23:1266. doi: 10.1186/s12885-023-11734-0 (PMC10740344; doi:10.1186/s12885-023-11734-0)
Supplement: Supplementary file 4 — Additional file 4: Supplementary material 4. Strategies to achieve rigour25. [file 12885_2023_11734_MOESM4_ESM.docx]

| **Rigour criterion** | **Strategy** | **Steps taken to achieve rigour** |
| --- | --- | --- |
| Credibility | Investigators’ authority | - Interviewers had the knowledge and skills to perform their roles.  - Interviewers were guided by senior investigators in both the field of early rectal cancer and qualitative research. |
|  | Prolonged engagement | - Researcher had several years of experience in research of the specific subject and were familiar with frequent concerns of patients and healthcare providers in the clinical decision-making process of early rectal cancer. |
|  | Peer debriefing | - Research meetings were held with the project group to discuss and assess findings. |
| Dependability | Description of study methods | - An interview guideline and topic list were established. |
|  | Audit trail | - Data collection was tracked throughout the data collection process. |
|  | Auditing | - Coding accuracy and intercoder differences were discussed between the investigators. |
| Confirmability | Triangulation | - Several types of triangulation including data and investigator triangulation were applied. |
|  | Audit trail | - Interviews were recorded and transcribed according to the recording. |
|  | Reflexivity | - After the interviews, the interviewers discussed how their experiences, potential bias and feelings may have influenced the interviews and assessment of data. |
| Transferability | Sampling | - A purposeful sample size was invited to participate. |
|  | Data saturation | - Audio was recorded and data saturation was reached for both patients and healthcare providers. |

**Supplementary material 4. Strategies to achieve rigour^25^.**
